# Assessment of malaria as a public health problem in and around Arjo Didhessa sugar cane plantation area, Western Ethiopia

**DOI:** 10.1186/s12889-020-08784-5

**Published:** 2020-05-12

**Authors:** Mebrate Dufera, Regea Dabsu, Gemechu Tiruneh

**Affiliations:** 1 Department of Biology, College of Natural and Computational Sciences, Wollega University, Post Box No: 395, Nekemte, Ethiopia; 2 Department of Medical Laboratory Sciences, Institute of Health Sciences, Wollega University, Post Box No: 395, Nekemte, Ethiopia

**Keywords:** Arjo Didhessa, Malaria, Malaria risk factors

## Abstract

**Background:**

Although much progress has been made in reducing malaria morbidity and mortality worldwide in the last decade, nationally malaria remains the third leading cause of death and still considered a major public health problem. Therefore, this study was aimed to assess malaria as a public health problem in and around the sugar cane plantation area of Arjo Didhessa sugar factory, Western Ethiopia.

**Methods:**

A community-based cross-sectional study supplemented with clinical retrospective data, which included 452 study subjects was recruited and the study period was extended from May 2016 up to November of 2017. A standardized questionnaire was used to assess malaria risk factors and blood samples were received from all study participants and further subjected to Giemsa staining for determination of malaria prevalence. Data were analyzed by SPSS version 20. Malaria risk factors were identified by multivariate logistic regression at a significance level of *P < 0.05*.

**Results:**

The overall malaria prevalence was 3.1%; *Plasmodium vivax* is the main type of malaria parasite. Overnight outdoor sleeping and improper utilization of mosquito bed nets were found to be statistically significant as malaria risk factors in the community. In the retrospective studies of five years, the peak malaria cases (13.84%) were reported in 2013 and the lowest cases (1.24%) in 2017.

**Conclusions:**

The figure for malaria witnessed in this area remains higher than the observed national malaria prevalence indicating malaria remains a public health problem. Therefore, we suggest the factory administrators and health care professionals work more on raising awareness to avoid night outdoor sleeping and promote frequent and appropriate utilization of insecticide-treated nets in line with regular indoor residual spraying.

## Background

Malaria is a haemoparasitic disease caused by obligate intracellular protozoan parasites of plasmodium species which are transmitted by infected female anopheline mosquito. Among the five types of plasmodium parasites that cause malaria, *Plasmodium vivax* and *Plasmodium falciparum* are widely distributed in Ethiopia and worldwide [1].

Irrespective of the promising strives made so far, to reduce malaria-related mortality and morbidity, malaria is the third leading cause of mortality next to HIV/AIDS and TB among infectious diseases. Therefore, malaria is considered one of the still existing health threats causing a considerable amount of mortality, morbidity and economic burden affecting all parts of the sub-Saharan African countries in which the problem is aggravated [2].

As indicated in the World malaria report of 2018, a promising effort was made to combat malaria. The success to tackle the disease was lowered during the years of 2015 through 2017 in which, in 2017 a total of 219 million and 435,000 malaria cases and malaria attributed deaths were reported respectively [3].

Ethiopia is among the countries with a large burden of malaria with the peak transmission rate in the world. According to the survey of indicators’ of malaria taken in Ethiopia in 2015, overall malaria parasite prevalence is 0.5% in a population residing in malarious areas and a total of 2,174,707 malaria cases were detected and (63.7%) of these cases were *Plasmodium falciparum* [4].

More than half (60%) of Ethiopia’s population lives in malarious areas, and 68% of the country’s landmass is favorable for malaria transmission. Malaria transmission occurs throughout the year with the highst transmission period from June to September which is considered as a major transmission season in the country [4].

Transmission is mostly geo-spatially heterogeneous throughout the year and among the years. Malaria epidemics occur every five to eight years in the country. Social and natural factors mark the transmission scheme of malaria. Temperature, relative humidity, and rainfall are the key natural features that influence the breeding character of mosquito and malaria parasites [5, 6].

Malaria parasite favor’s increased humidity indices for completion of its major life cycle phases. Researches addressing prevalence helps to assess malaria status within a given locality and has an important indication to value the overall effectiveness of prevention strategies being implemented in the area [6, 7].

About half of a population living in areas of an altitudinal range of 1500 and 2500 m above sea level are more likely to get malaria and these areas experience hit in malaria outbreak in Ethiopia Some studies from high altitude zones identified age, the proximity of households to potential mosquito breeding sites, sharing of houses with cattle, presence of windows and open attics as malaria risk factors. In addition to this, malaria is also related to factors like altitude, rainfall, and temperature. Thus, interventions focus on both the households and the surrounding environment [5, 7].

In Africa, members of *Anopheles gambiae* complex and *Anopheles funestus* are widely distributed and are causes for the spread of malaria in the region. *Anopheles gambiaes*.s is the most anthropophagic species of malaria vector with characteristic indoor and outdoor resting. *Anopheles arabiensis* and *Anopheles quadriannulatus* species are one of the species of the *Anopheles gambiae* complex that are found in Ethiopia [8].

Entomological findings conducted so far indicated the presence of 42 anopheles in Ethiopia. Despite the presence of all these, only *Anopheles arabiensis* is known to play a major contribution in the spread of malaria in the country. Others like *Anopheles funestus* and *Anopheles pharoensis* playing a secondary role, while *Anopheles nili* involves transmission in localized areas [9].

WHO has initiated strategies to control malaria in 1992. Since that time, emphasis on malaria control has shifted from vector eradication to increased case detection and treatment Efforts to control malaria include environmental management, insecticide sprays and use of Insecticide-treated nets (ITNs) [6, 10]. In Ethiopia, the key malaria control strategies are prompt diagnosis and immediate treatment of cases. Besides these, there are other strategies like outbreak investigation and arrest, mosquito vector control and environmental management. Indoor residual sprays and insecticide-treated nets are also used at a large [10].

Unstable malaria transmission occurs in Ethiopia and makes the country vulnerable to focal and multifocal devastating malaria outbreaks. Malaria is most of Ethiopia is mainly characterized by its seasonality. The transmission intensity and prevalence pattern variably differs with ranging altitude, temperature, and social mobility. Control of the disease is stepped on key universal strategies, such as prompt and proper case management, intermittent preventive treatment (IPT) during pregnancy and integrated vector management (IVM) encompassing the use of insecticide-treated nets (ITN), indoor residual spraying (IRS), and environmental management [2].

According to the retrospective trend analysis of malaria cases done in Ataye District Hospital, 31,810 blood films examined from malaria suspected patients from January 2013 to December 2017. Among these blood films, 2670 (8.4%) were microscopically ascertained malaria cases. In 2016, a higher number (8066) of malaria suspected patients were examined and 863 (10.7%) of them became microscopically confirmed cases. On the other hand, out of 6172 malaria suspected patients, the least number of cases, 358 (5.8%), were recorded in 2017. Generally, malaria soared during the years 2013 through 2016 and declined in 2017 [11].

A ten-year retrospective malaria trend analysis conducted in Sibu-Sire, western Ethiopia, from 2004 to 2013, demonstrated that among a total of 30,070 blood films requested for malaria diagnosis, 6036 (20.07%) microscopically diagnosed malaria parasites recorded which gives an average of 603.6 malaria cases. No year reported zero malaria cases. The lowest rate (1.6%) malaria cases recorded in 2008 and the highest (31.2%) in 2004, followed by 2010, 2005 at a prevalence of 13.7 and 13%, respectively. Furthermore, malaria rose in all months of the year with different fluctuation rate in which, the highest peak was in June at a prevalence rate of 18.9%, followed by May, November, and July with a prevalence of 13.3, 13.2, and 11.2%, respectively [12].

To our knowledge level, the present study is the first community-based malaria survey in the vicinity and can be considered as a baseline survey which would help provide information and fill the knowledge gap regarding malaria prevalence, predictors of malaria prevalence and the fluctuating trend of malaria observed over the years around the area. Thus this study was designed to assess the prevalence of malaria and its associated factors in and around Arjo Didhessa sugar factory, Western, Ethiopia.

## Methods

### Study design and setting

A community-based cross-sectional survey study was designed to determine the current malaria prevalence and its associated factors in and around Arjo Didhessa sugar factory, Western, Ethiopia. Additionally, a retrospective 5-year (2013–2017) malaria trend analysis was designed at Arjo- Didhessa health center to determine malaria cases. The study was undertaken amongst sugar cane workers or laborers due to their seasonal activities and outdoor sleeping behaviors without bed nets. The health center provides a general health service in addition to malaria control and treatment for the catchment population. In and around Arjo Didhessa sugar cane plantation area, five study clusters (Abote Didhessa, Command 2, Command 5, Command 8 and main camp) were purposefully selected and used for the survey study.

### Study area

The research was undertaken in five study clusters found in and around Arjo Didhessa sugar factory from May–September 2017. Four study clusters are found in the sugar factory and the remaining one surrounding the factory. The area is located at Western Ethiopia of Oromia Regional State in east Wollega, Ilu Ababora and Jimma Zones at the Didhessa Valley at a distance of 540 km from the capital through the route of Addis Ababa-Jimma-Beddele-Nekemet Road. The study area has generally a lowland climate with an altitudinal range of 1570–1275 masl. The mean annual rainfall is 801–1400 mm. Maize, *Eragrostis teff* and pepper is cultivated for food and income. A small-scale cattle breeding also exists. The Factory in total has 20,000 ha of land cultivated with sugar cane. The factory has 800 permanent and 1000 temporary workers. Sixty-four residential houses, one functional health center and two service giving buildings intended to offer common dining and recreation services. This inevitably leads to the attraction of more labor force to this irrigated area.

### Sample size determination and study subjects

Calculation of sample size was done using the formula for estimating single proportion (*n* = Z^2^ P (1-P) /d2), Where *n* = sample size d = worst accepted value/marginal error, Z = is statistic value for level of 95% confidence, is 1. 96; *P* = is expected prevalence or proportion which is 0.5. However, since there were no previous or pilot malaria studies conducted in the area and data from the clinic were studied only after the epidemiological study was done, for the survey study 50% was assumed for prevalence. A minimum of 384 samples was generated using a 5% marginal error. Once the minimum number of samples was obtained to get the largest sample size, 17% contingency was added and 452 study subjects of both sexes aged 5 years and above were enrolled in the survey study. All randomly selected household heads and family members in the selected study clusters of Arjo Didhessa sugar cane plantation area were the source of the study population for the interview, questionnaire, and parasitological blood film investigation respectively. A pre-coded questionnaire was deployed to interview the family heads living in the selected households in a face- to- face interview approach. A blood sample was collected from a finger of each member in the selected households for a smear test. All household heads and family members who were available in the selected households during sample collection were eligible for interview and smear tests. However, relatives who came during the study and family members who were not available in the home were excluded.

### Data collection procedures

#### Malaria parasite microscopy

All 452 individuals were requested to provide a capillary blood sample from fingertip immediately after the interview, for parasitological examination. Thick and thin blood films were made separately on a single glass slide. The procedure was performed by a trained medical laboratory technician as per the standard [13]. The slides were labeled, air-dried and the thin film was fixed with methanol before staining with Giemsa. Slides were subjected to 3% Giemsa for 30–45 min at Arjo Didhessa sugar factory health center laboratory unit. Blood slides were read by 100X objective lens of an Olympus microscope. A minimum of 200 fields was scanned to report negative slides. After cross-checking, the slides were reported as either negative for blood parasites, *P. falciparum* positive, *P. vivax* positive or mixed infection with both *P. falciparum* and *P. vivax* [14]. All slides were cross-checked blindly by independent microscopist and concordant results were reported as a final result.

#### Structured questionnaire survey

For the cross-sectional survey study, a structured questionnaire addressing socio-demographics, household characteristics and health behavioral factors and other duty categories of the residents were used. The survey questionnaire was based on the malaria indicator survey household questionnaires, which were filled by the participants. Before commencing data collection, the tool was piloted among 5% of the sample in nearby localities not involved in the actual data collection. A total of ten data collectors and two supervisors took part in data collection after receiving 2 days of exhaustive training. Besides, the investigators were responsible for offering the training and monitoring of the overall data collection activities. The questionnaire was administered to 452 volunteers by trained interviewers considering the schedule of the participants.

#### Retrospective health facility data

In Arjo Didhessa sugar factory health center, peripheral blood is routinely examined for malaria parasite detection according to the standard operating procedure of malaria in Ethiopia. Data were retrieved on malaria in the past 5-year (2013–2017) from the health service laboratory unit registry to compute the trend of malaria in the community. Specific data was extracted on species of malaria identified, total cases suspected of malaria, annual, monthly and seasonal cases of malaria.

#### Data entry and analysis

The data were checked for completeness and consistency and entered (twice) into a statistical program for social sciences version 20.0 (IBM Corp., Armonk, N.Y., USA). A descriptive analysis was computed for all variables. Association between the independent and dependent variables was measured and tested using OR and 95%CI. Binary logistic regression was used to build the fitting model for multivariate analysis. Candidate variables were selected for multivariate analysis based on purposeful selection of the variables at *P* = 0.25 in the univariate analysis. The significant level was considered at *P* < 0.05 in the multivariate model. Odds ratio (OR) at a 95% confidence interval was considered to see the association between the prevalence of malaria and the independent variables. The prevalence of malaria is considered as the main outcome variable in the analysis. All variables with a crude odds ratio having a *p*-value of less than 0.2 were transferred to the final adjusted model.

## Results

### Socio-demographic characteristics of the study participants

A total of 452 study participants were surveyed for the cross-sectional study. A majority of them, (67.9%), were males with a mean age of 26.5 and a standard deviation (SD) of 12. Around 46% of the respondents were daily laborers and about 73% have no formal educational background or were educated up to the primary cycle concerning educational attainment. Half of the surveyed residents were married and the remaining portion was single. A significant number (60%) of the respondents were dwelling in a conventional type housing unit. A majority, (70%), of the total respondents, lived in the surrounding area for at least 5 years or more. Furthermore, the study site was divided into five clustering segments which contributed a comparable amount of study participants of which the highest number 132 (30%) was chosen from ‘Abote Didhessa’ clustering unit (Table 1).

**Table 1:** Characteristics of socio-demographic, socio-economic and housing condition of study participants living in and around Arjo Didhessa sugar cane plantation area (May–November, 2017)

Variables (*n* = 452)	Category	Frequency n (%)
Sex	Male	307 (67.9%)
	Female	145 (32.1%)
Age	Age less than 15 years	32 (7.1%)
	Age 15–30 years	313 (69.2%)
	Age greater than 30 years	107 (23.7%)
Occupation	Daily laborer	208 (46.0%)
	Farmer	117 (25.9%)
	Government employee	83 (18.4%)
	Others^a^	44 (9.7%)
Educational Status	No formal education/read &write only	166 (36.7%)
	Primary education	165 (36.5%)
	Secondary education	70 (15.5%)
	Tertiary education	51 (11.3%)
Marital Status	Single	234 (51.8%)
	Married	218 (48.2%)
Duration In The Village	Stay <= five years	316 (69.9%)
	Stay more than 5 years	136 (30.1%)
Study clusters	Abote Didhessa	132 (29.2%)
	Command 2	74 (16.4%)
	Command 5	90 (19.9%)
	Command 8	64 (14.2%)
	Main camp	92 (20.4%)
Housing Unit	Conventional	268 (59.3%)
	Improved	42 (9.3%)
	Others^b^	142 (31.4%)

Keys ^a^ Students, housewives, merchants

^b^ Housing units made walls of iron bars

### Prevalence of malaria

Among the 452 surveyed participants, a blood sample was taken from 443(98%) assented individuals for parasitological examination and a total of 14 laboratory-confirmed malaria parasites were found to exist giving an overall malaria prevalence of 3.1% around the sugar factory during the study period. Like the other remaining parts of Ethiopia, only the two major species of malaria; *Plasmodium vivax* 8 (57%) and *Plasmodium falciparum* 6 (43%) were detected during the survey. No significant difference was observed regarding malaria distribution among the four clustered communities but more number of malaria parasites was detected in blood samples of respondents from Abote Didhessa and all individuals with malaria case were living in a conventional housing type unit (Table 2).

**Table 2:** Prevalence of malaria among study participants living in and around Arjo Didhessa sugar cane plantation area (May–November, 2017)

Variables (***n*** = 452)	Category	Number of surveyed participants	Number of malaria parasite detected per category	Prevalence of malaria per category (%)
**Sex**	Male	307	13	4.23%
	Female	145	1	0.68%
**Age**	Age less than 15 years	313	10	3.2%
	Age 15–30 years	107	2	1.87%
	Age greater than 30 years	32	2	6.25%
**Occupation**	Daily laborer	208	5	2.40%
	Farmer	117	4	3.42%
	Government employee	83	3	3.61%
	Others^a^	44	2	4.55%
**Marital Status**	Single	234	8	3.42%
	Married	218	6	2.75%
**Duration In The Village**	Stay <= five years	316	8	2.53%
	Stay more than 5 years	136	6	4.41%
**Study Clusters**	Abote Didhessa	132	6	4.5%
	Command 2	74	3	4.05%
	Command 5	90	1	1.1%
	Command 8	64	1	1.56%
	Main camp	92	3	3.26%
**Housing Unit**	Conventional	268	14	5.2%
	Improved	42	0	0%
	Others	142	0	0%
**Parasite species**	*Plasmodium vivax*	452	8	1.8%
	*Plasmodium falciparum*	452	6	1.3%
	Total malaria parasite	452	14	3.1%^b^

Key ^a^Students, housewives, merchants

^b^Overall prevalence of malaria

### Factors associated with the prevalence of malaria

A binary logistic regression model was used to identify factors associated with malaria prevalence in the vicinity. In the bivariate model, all variables were included to identify candidate variables fitting to the final model of multivariate analysis. Among the surveyed individuals, three variables were found to become predictors of malaria prevalence. Individuals who practiced sleeping outdoor were seventy-seven times more likely to acquire malaria when compared to indoor sleeping counterparts (AOR, 77 (8–78.9). In the study area, indoor residual spraying and bed net utilization behavior were among the variables negatively associated with existing malaria prevalence. Among the surveyed groups those individuals who do not utilize bed nets in their home have eleven times the odds of developing malaria (AOR, 11(2–65) than those who used bed nets frequently. On the other hand, individuals who were absent during spray time have fourteen times odds to develop malaria than who have sprayed their home (AOR, 14(1.3–15.8) (Table 3).

**Table 3:** Factors associated with malaria prevalence among study participants living in and around Arjo-Didhessa sugar cane plantation area (May–November, 2017)

Variables (***n*** = 452)	Category	Crude Odds ratio COR (95%CI)	***P***-value	Adjusted Odds ratio AOR (95%CI)	***P***-value
**Sex**	Male	referent		referent	
	Female	6.4 (0.8–49)	0.076		
**Presence of livestock**	yes	2.9 (0.8–9.5)	0.080		
	no	referent		referent	
**Sleep outdoor**	yes	20.8 (6.2–68.9)	< 0.001	77 (8–78.9)	0.005^
	no	referent		referent	
**Previous malaria history**	yes	3.138 (0.8–11)	0.082		
	no	referent			
**Presence of damp/stagnant water**	yes	17 (4.5–60)			
	no	referent	0.01	referent	
**Treatment with anti-malaria drugs**	yes	4.18 (0.8–21.5)	0.087		
	no	referent		referent	
**Bed net coverage**	yes	Referent			
	no	4 (1.2–13.3)	0.025		
**Bed net utilization**	yes	Referent		referent	
	no	22 (7–70)	< 0.001	11 (2–65)	0.005^
**Bed net utilization target**	All	Referent		referent	
	Fathers and mothers	1.5 (0.18–12)	0.12		
	children	11.5 (2–63.5)	0.005		
**Indoor Chemical spray**	Yes	Referent		referent	
	No	5.84 (1.9–17.2)	0.001	14 (1.3–15.8)	0.026^

Key ^ statistically significant variables at *p*-value =0.05 < 0.05

### The retrospective trend of malaria

Regarding the retrospective study of annual trends of malaria cases, within the last five successive years (2013–2017), in Arjo Didhessa sugar cane plantation area a total of 65,275 patients visited the prime public health center were found around the sugar factory. Among these patients, there were a total of 4164 laboratories confirmed malaria cases which yielded an estimated malaria case proportion of 6.38% and mean annual malaria cases of 832.8. During the 5 years (2013–2017), the retrospective clinical data revealed a slight fluctuating trend of malaria occurrence. The peak malaria case occurrence was in 2013 (13.84%) and the lowest malaria occurring year was in 2017 (1.24%) showing a remarkable reduction in 2017. In general, the trend of malaria observed over the 5 years exhibited a peak incidence in the year 2013 and 2015 and then a steady fall in 2016 and 2017. Malaria trend analysis was not described for the ‘Abote Didhessa’ clustering unit, which is attributed to the lack of consecutive retrospective clinical data. The dominant types of plasmodium species were *Plasmodium vivax* 3170 (4.85) over the 5 years (Table 4).

**Table 4:** Annual malaria trend case proportion in and around Arjo Didhessa sugar cane plantation area (2013–2017)

Year	Total patients visited OPD	Case proportion No (%)			
		*P. falciparum*	*P. vivax*	Mixed (*P*. *falciparum + P. vivax*)	Total (% trend prevalence)
2013	4234	235 (5.55)	307 (7.25)	44 (1.04)	586 (13.84)
2014	11,234	260 (2.31)	1081 (9.62)	61 (0.54)	1402 (12.48)
2015	15,318	200 (1.31)	1317 (8.6)	100 (0.65)	1617 (10.56)
2016	16,928	44 (0.26)	287 (1.7)	10 (0.06)	341 (2.01)
2017	17,561	37 (0.21)	178 (1.012)	3 (0.02)	218 (1.24)
Total	65,275	776 (1.19)	3170 (4.85)	218 (0.33)	4164 (6.38)

**Fig. 1:**
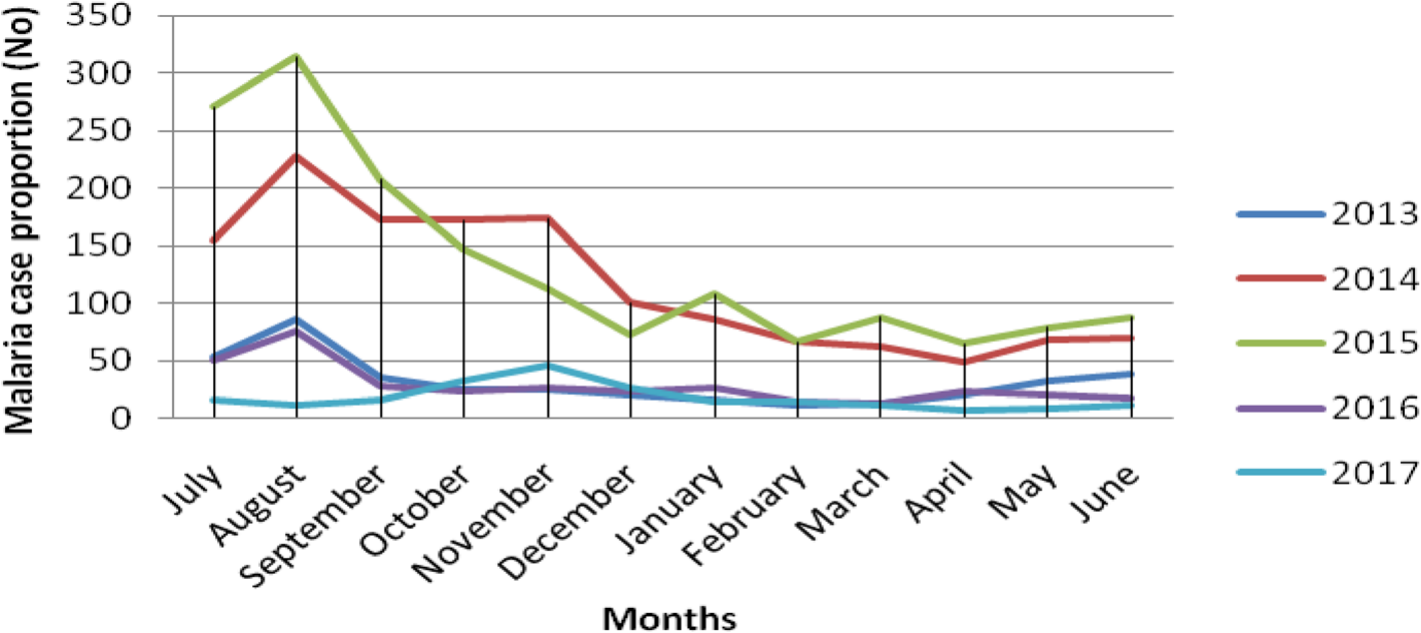
The five years analyses of malaria trend; Malaria case proportion based on month and year in and around Arjo Didhessa sugar cane plantation area (2013–2017)

**Fig. 2:**
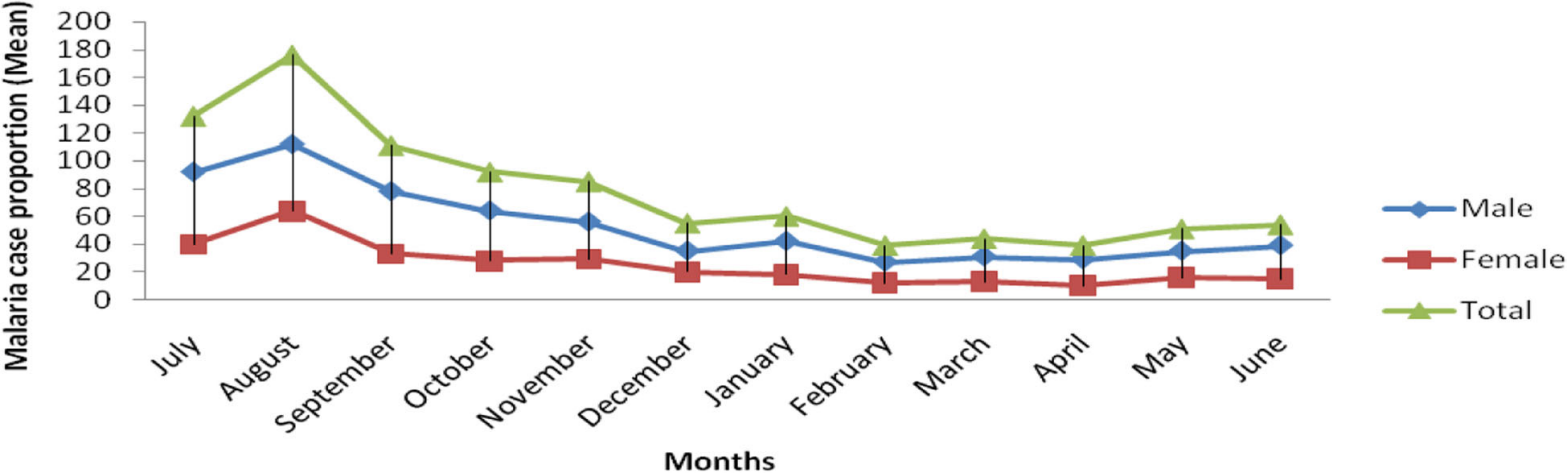
The five years analyses of malaria trend; Mean monthly malaria case proportion by sex in and around Arjo Didhessa sugar cane plantation area (2013–2017)

In the present study, the monthly malaria trend case proportion indicated that the peak cases were observed in August for each year which lies within the summer season (June–August) of Ethiopia. The highest case of malaria seen in August 2015 which was 314 cases and the lowest was in April 2017 with only 7 malaria cases (Fig. 1). Although it was not statistically supported, malaria cases were higher in males throughout all the months of the years in the retrospective study (Fig. 2).

Concerning the identified Plasmodium species, both species of plasmodium reported in each year with *Plasmodium vivax* is the predominant species used to be reported in the study area. *Plasmodium vivax* accounted for 76.12% and *Plasmodium falciparum* 18.63% mixed (both *Plasmodium falciparum* and *Plasmodium vivax*) 5.23% of the total malaria cases. Both species seemingly decreasing uniformly every year and no fluctuation and trend shift was observed.

## Discussions

In this study, the investigators tried to assess the overall impact of malaria as a public health problem in and around Arjo Didhessa sugar cane plantation areas. Malaria prevalence and malaria risk factors were assessed using a community-based cross-sectional study design and also malaria trend analysis was depicted in the area 5 years before the study.

Among a total of 452 study participants, a blood sample was received from 443 in which the overall active malaria prevalence yielded during the survey was 14 (3.1%). Of the total malaria parasite detected, *Plasmodium vivax* accounts 8 (57%) and the remaining type 6 (43%) is *Plasmodium falciparum*. The community prevalence of malaria explored in the current study is comparably similar to the findings of the study conducted in the Fincha sugar factory, located at Western Ethiopia which reported an overall malaria prevalence of 2.6% [15]. Similarly, the study is in line with the findings of another study carried out in the central part of Ethiopia that reported 4.2% [16]. The present study is also in agreement with the previous study in Gedeo Zone, Southern Ethiopia, in which the identified dominant species is *Plasmodium vivax* followed by *Plasmodium falciparum*, but in contrast with the overall prevalence (16%) [14]. In the present finding, there was no mixed infection reported which is comparable with a study done in Metema Hospital in which the mixed infection is only 0.3% [17]. The finding of the current study was also in concurrence with another study done in Sudan which reported a 3.8% prevalence of concomitant malaria [18].

According to the 2015 national malaria indicator survey, the study area falls among highland fringes and moderate transmission risk woredas of Ethiopia with API (Annual Parasite Index) of 0–5 [2]. This result is much higher than the result of a national malaria indicator survey which is 0.55%. This disparity could be because the study area apart from its geographical location in malarious areas is a newly established plantation site and presence of irrigation. Irrigated areas are potentially creating a conducive environment for the reproducibility of malaria vector which makes prone the area to a relatively higher percentage of malaria prevalence and also the survey was held at a peak time of malaria transmission period.

Concerning risk factors, individuals who practiced or who have had an experience of outdoor sleeping during night time were seventy-seven times more likely to acquire malaria when compared to indoor sleeping counterparts (AOR, 77 (8–78.9). Furthermore, bed net utilization behavior and indoor residual spraying were among the variables negatively associated with the existing malaria prevalence in the study area. Among the surveyed groups, individuals who did not utilize bed nets in their homes were eleven times more likely to develop malaria (AOR, 11(2–65) than those who used bed nets frequently. This is in line with a previous study in which outdoor sleeping and bed net utilization was associated with the risk of malaria (*P* < 0.05) West Armachiho District, Northwest Ethiopia [19]. There is evidence that workers came from relatively non-malarious areas to the factory and their mobility was based on the condition of activities and season of sugar cane plantation. In general, as it was suggested by previous studies conducted in Cambodia [20], a more focused type of intervention package is required which suits and best addresses the mobile population coming to the area.

In the present study, the trend of malaria indicated that the peak cases were observed in the 2013 and cases reduction in 2014 and 2015 and a steady fall in 2016 and 2017. This is in agreement with a seven-year retrospective study from Metema Hospital, Northwest Ethiopia in which a positive rate of malaria within the last 7 years (2006–2012) was almost constant with a slight fluctuation [17].

In the present study, the peak monthly cases were observed in August for each year which lies within the summer season (June–August) of Ethiopia. This is also similar to a study conducted in western Ethiopia [12]. But the other two studies reported that the retrospective trend of malaria shows peak cases of malaria were reported during the winter season from September–December [11, 12]. Malaria cases were higher in males in the retrospective malaria trend of this study. This result also agrees with other studies that reported a retrospective malaria trend in their analysis [11, 12, 17].

Even though the trend of malaria is moving down, the prevalence of malaria observed in the community is still higher than the yearly malaria case proportion seen at the clinic last year. This rise is owing to the period of the data collection being the major malaria transmission period.

### Limitations of the study

Although the investigators tried to assess a relatively wide range of malaria situation, focusing on current status supplemented with cumulative prevalence in the past, the study was not rid of limitations. The main limitation of the study was incompleteness and inconsistency of retrospective data. In the retrospective part of the study, only the total malaria cases over the 5 years were presented not the prevalence of malaria.

The diagnosis of malaria was used to be reported by microscopy in the health center during the past 5 years before the study. If this method was missing or malfunctioning, the other technique, which is malaria rapid test, was used as an alternative method. Hence, for our consumption, we reported the data arising from a combination of both techniques of diagnosis. While calculating the trend analyses, some important variables like age and residence were not included because of the incompleteness of the data in some specific years.

## Conclusion

The figure for malaria witnessed in this area remains higher than the observed national malaria prevalence. As Ethiopia is aspiring to eliminate malaria, intense efforts are further needed within factories that attract a large number of daily laborers, especially in malaria-endemic areas. The factory has irrigational farming of sugarcane plantation this, in turn, makes the area a potential site for malaria vector breeding and malaria remains a public health problem. Therefore, we suggest the factory administrators and health care professionals work more on raising awareness to avoid night outdoor sleeping and effective and appropriate utilization of insecticide-treated nets. It is recommended that the factory has to enhance regular indoor residual spraying to reach complete spray coverage within the factory and the area as well.

## Data Availability

The data set generated from patients’ clinical records is not publicly available to protect patient confidentiality. Unidentifiable data can be obtained from the corresponding author upon reasonable request.

## Abbreviations

AIDS: Acquired immune deficiency syndrome
AOR: Adjusted odds ratio
API: Annual parasite index
COR: Crude odds ratio
HIV: Human immune deficiency virus
IPT: Intermittent preventive treatment
IRS: Indoor residual spraying
ITN: Insecticide treated net
IVM: Integrated vector management
OPD: Out-patient department
SD: Standard deviation
SOP: Standard operating procedure
TB: Tuberculosis
WHO: World health organization
